# Sickness and the New Poor

**Published:** 1920-12-04

**Authors:** 


### Sickness and the New Poor.

An interesting article, contributed by Mrs. Mary C. D. Walters, appears in a recent number of *The Woman's Leader*, in which the writer discusses the problem confronting the "new poor" in time of sickness. "What do we mean," she asks, "when we speak of the new 'poor'? It is time for it to be realised in a practical way, that poverty, as it was formerly understood among the artisan classes, hardly exists, and that the burden of poverty lies heaviest now upon the salaried workers, upon the very class who, in the days of their comparative affluence, were the most generous, although the least ostentatious, supporters of the hospital, and yet who, in their day of need, have practically no claim on its services? This class, the 'new poor,' as they have come to be called, includes those whose work we can as little dispense with as with that of the artisan. It includes the professional and business man and his wife, upon whose capacity and health depend the well-being of the whole household.

"It includes the bachelor whose means are such that he does not dare, under present conditions, to undertake the responsibilities of marriage, and it includes the single woman worker who depends entirely upon her own efforts not only for her support now, but for making provision for her old age. Last, but surely not least, it includes those of advanced age who are, owing to quite unforeseen circumstances, living on incomes all too slender for their increasing needs, or who are being supported in whole or in part by sons or daughters, with heavy burdens of their own. . . .

"Many middle-class people insure themselves against accident and disease, thus providing themselves with a small sum of money towards the expenses involved, but this does not facilitate home-nursing. Could the 'new poor,' who helped their needier brothers and sisters of yore by a yearly subscription to the hospital fund, know that such subscriptions would now secure for them what they formerly bestowed on others, how willingly would they pay it now; the hospital would be the gainer, for it should be entitled to charge the now comparatively well-to-do artisan a small sum for the attendance he has up till now received free."

Almost immediately after this article was brought to our notice we received a pathetic letter from one of these "new poor," a lady over fifty years of age, who writes of her practical difficulties and future anxieties, particularly of the haunting fear lest she should be overtaken by illness, and concludes thus: "The fact remains that while it costs many a middle-class woman all she can earn in health, she dreads the idea of sickness and the infirmities of old age. *The State will have to deal with it, or else provide licensed lethal chambers for a decent exit.*"

It is a hard saying in the twentieth century!

